# Supplementary material for: Assessment of the consistency of health and demographic surveillance and household survey data: A demonstration at two HDSS sites in The Gambia
Source: PLoS One. 2022 Jul 13;17(7):e0271464. doi: 10.1371/journal.pone.0271464 (PMC9278757; doi:10.1371/journal.pone.0271464)
Supplement: S3 File — (PDF) [file pone.0271464.s003.pdf]

```
--------------------------------------------------------------------------------
      name:  <unnamed>
       log:  C:\Users\mjasseh\......\Final Version\PLoS ONE Re-submission\Response
to Queries\Kaplan-Meier List.smcl
  log type:  smcl
 opened on:  19 Apr 2022, 15:54:44

. sts list if year>10 & site=="FFN" & survey==0, by(per) failure
risktable(.0793977 1 5)

         Failure _d: endevent==1
   Analysis time _t: (enddate-origin)/365.25
             Origin: time dob
  Enter on or after: time startdate
        ID variable: id

Kaplan–Meier failure function
By variable: per

             At           Net     Failure      Std.
  Time     risk   Fail   lost    function     error     [95% conf. int.]
-----------------------------------------------------------------------
2001-2005
 .0794     2242     39     90      0.0277    0.0034     0.0217    0.0352
     1     2113     57    465      0.0450    0.0043     0.0373    0.0544
     5     1591      0   1591      0.0738    0.0056     0.0635    0.0857
2006-2010
 .0794     3302     33    173      0.0264    0.0028     0.0216    0.0324
     1     3096     36    887      0.0364    0.0032     0.0306    0.0433
     5     2173      0   2173      0.0490    0.0038     0.0421    0.0571
2011-2015
 .0794     3763     37    -41      0.0318    0.0028     0.0268    0.0377
     1     3767     53    483      0.0413    0.0032     0.0356    0.0480
     5     3231      0   3231      0.0553    0.0036     0.0486    0.0629
-----------------------------------------------------------------------
Notes: Survival-time summaries shown for specified times in (0, 5].
       Net lost equals the number lost minus the number who entered.




. sts list if year>10 & site=="FFN" & survey==1, by(per) failure
risktable(.0793977 1 5)

         Failure _d: endevent==1
   Analysis time _t: (enddate-origin)/365.25
             Origin: time dob
  Enter on or after: time startdate
        ID variable: id

Kaplan–Meier failure function
By variable: per
```

```
             At           Net     Failure      Std.
  Time     risk   Fail   lost    function     error     [95% conf. int.]
------------------------------------------------------------------------
2001-2005
 .0794     2510     53   -115      0.0174    0.0026     0.0130    0.0233
     1     2572    102    -40      0.0378    0.0038     0.0311    0.0459
     5     2510      0   2510      0.0755    0.0051     0.0660    0.0863
2006-2010
 .0794     3964     49    -57      0.0100    0.0016     0.0074    0.0136
     1     3972     75    359      0.0221    0.0023     0.0180    0.0272
     5     3538      0   3538      0.0409    0.0031     0.0352    0.0475
2011-2015
 .0794     4689     54   -140      0.0139    0.0017     0.0110    0.0177
     1     4775     83    198      0.0251    0.0023     0.0210    0.0299
     5     4494      0   4494      0.0421    0.0029     0.0367    0.0481
------------------------------------------------------------------------
Notes: Survival-time summaries shown for specified times in (0, 5].
       Net lost equals the number lost minus the number who entered.

. sts list if year>10 & site=="BAS" & survey==0, by(per) failure
risktable(.0793977 1 5)

         Failure _d: endevent==1
   Analysis time _t: (enddate-origin)/365.25
             Origin: time dob
  Enter on or after: time startdate
        ID variable: id

Kaplan–Meier failure function
By variable: per

             At           Net     Failure      Std.
  Time     risk   Fail   lost    function     error     [95% conf. int.]
------------------------------------------------------------------------
2001-2005
 .0794     2018     55     85      0.0288    0.0037     0.0224    0.0369
     1     1878     72    411      0.0558    0.0051     0.0467    0.0666
     5     1395      0   1395      0.0959    0.0067     0.0836    0.1099
2006-2010
 .0794     2916     52    154      0.0182    0.0024     0.0139    0.0236
     1     2710     59    745      0.0366    0.0035     0.0304    0.0441
     5     1906      0   1906      0.0599    0.0046     0.0516    0.0695
2011-2015
 .0794     3540     39     41      0.0179    0.0022     0.0141    0.0228
     1     3460     74    573      0.0291    0.0028     0.0241    0.0352
     5     2813      0   2813      0.0508    0.0037     0.0440    0.0586
------------------------------------------------------------------------
Notes: Survival-time summaries shown for specified times in (0, 5].
       Net lost equals the number lost minus the number who entered.
```

```
. sts list if year>10 & site=="BAS" & survey==1, by(per) failure
risktable(.0793977 1 5)

         Failure _d: endevent==1
   Analysis time _t: (enddate-origin)/365.25
             Origin: time dob
  Enter on or after: time startdate
        ID variable: id

Kaplan–Meier failure function
By variable: per

             At           Net     Failure      Std.
  Time     risk   Fail   lost    function     error     [95% conf. int.]
-------------------------------------------------------------------------
2001-2005
 .0794        3      0      0      0.0000         .          .         .
     1        3      0     -1      0.0000         .          .         .
     5        4      0      4      0.0000         .          .         .
2006-2010
 .0794     6973    130   -308      0.0110    0.0012     0.0088    0.0138
     1     7151    257    663      0.0291    0.0020     0.0255    0.0333
     5     6231      0   6231      0.0654    0.0029     0.0598    0.0714
2011-2015
 .0794     9310    158   -488      0.0162    0.0013     0.0139    0.0190
     1     9640    259    872      0.0325    0.0018     0.0291    0.0362
     5     8509      0   8509      0.0590    0.0024     0.0545    0.0639
-------------------------------------------------------------------------
Notes: Survival-time summaries shown for specified times in (0, 5].
       Net lost equals the number lost minus the number who entered.
```

```
. sts list if year>10 & site=="FFN" & survey==0, by(year) failure
risktable(.0793977 1 5)

         Failure _d: endevent==1
   Analysis time _t: (enddate-origin)/365.25
             Origin: time dob
  Enter on or after: time startdate
        ID variable: id

Kaplan–Meier failure function
By variable: year

             At           Net     Failure      Std.
  Time     risk   Fail   lost    function     error     [95% conf. int.]
-------------------------------------------------------------------------
2001
 .0794      371      7   -108      0.0321    0.0091     0.0183    0.0558
     1      472     10    230      0.0478    0.0108     0.0307    0.0741
     5      232      0    232      0.0796    0.0144     0.0557    0.1132
2002
```

| | | | | | | | |
|---|---|---|---|---|---|---|---|
| .0794 | 385 | 7 | 21 | 0.0297 | 0.0084 | 0.0170 | 0.0517 |
| 1 | 357 | 14 | 56 | 0.0480 | 0.0108 | 0.0309 | 0.0743 |
| 5 | 287 | 0 | 287 | 0.0819 | 0.0138 | 0.0587 | 0.1137 |
| 2003 | | | | | | | |
| .0794 | 445 | 7 | 54 | 0.0281 | 0.0077 | 0.0164 | 0.0478 |
| 1 | 384 | 11 | 77 | 0.0444 | 0.0097 | 0.0289 | 0.0680 |
| 5 | 296 | 0 | 296 | 0.0720 | 0.0125 | 0.0511 | 0.1011 |
| 2004 | | | | | | | |
| .0794 | 457 | 10 | 4 | 0.0292 | 0.0077 | 0.0174 | 0.0488 |
| 1 | 443 | 14 | 108 | 0.0513 | 0.0102 | 0.0347 | 0.0756 |
| 5 | 321 | 0 | 321 | 0.0845 | 0.0132 | 0.0621 | 0.1144 |
| 2005 | | | | | | | |
| .0794 | 584 | 8 | 119 | 0.0220 | 0.0060 | 0.0128 | 0.0376 |
| 1 | 457 | 8 | -6 | 0.0369 | 0.0079 | 0.0242 | 0.0562 |
| 5 | 455 | 0 | 455 | 0.0543 | 0.0099 | 0.0379 | 0.0775 |
| 2006 | | | | | | | |
| .0794 | 523 | 8 | -55 | 0.0295 | 0.0073 | 0.0182 | 0.0477 |
| 1 | 570 | 1 | 221 | 0.0441 | 0.0088 | 0.0298 | 0.0652 |
| 5 | 348 | 0 | 348 | 0.0462 | 0.0090 | 0.0315 | 0.0677 |
| 2007 | | | | | | | |
| .0794 | 622 | 8 | 96 | 0.0142 | 0.0047 | 0.0074 | 0.0272 |
| 1 | 518 | 7 | 135 | 0.0284 | 0.0068 | 0.0177 | 0.0454 |
| 5 | 376 | 0 | 376 | 0.0426 | 0.0086 | 0.0286 | 0.0632 |
| 2008 | | | | | | | |
| .0794 | 702 | 2 | 83 | 0.0311 | 0.0064 | 0.0208 | 0.0465 |
| 1 | 617 | 9 | 175 | 0.0341 | 0.0067 | 0.0231 | 0.0500 |
| 5 | 433 | 0 | 433 | 0.0491 | 0.0083 | 0.0352 | 0.0682 |
| 2009 | | | | | | | |
| .0794 | 678 | 7 | -42 | 0.0256 | 0.0060 | 0.0162 | 0.0403 |
| 1 | 713 | 10 | 249 | 0.0353 | 0.0069 | 0.0240 | 0.0518 |
| 5 | 454 | 0 | 454 | 0.0504 | 0.0083 | 0.0364 | 0.0695 |
| 2010 | | | | | | | |
| .0794 | 777 | 8 | 91 | 0.0305 | 0.0061 | 0.0206 | 0.0452 |
| 1 | 678 | 9 | 107 | 0.0413 | 0.0072 | 0.0294 | 0.0579 |
| 5 | 562 | 0 | 562 | 0.0551 | 0.0084 | 0.0408 | 0.0742 |
| 2011 | | | | | | | |
| .0794 | 716 | 8 | -49 | 0.0215 | 0.0053 | 0.0133 | 0.0349 |
| 1 | 757 | 13 | 230 | 0.0322 | 0.0065 | 0.0217 | 0.0476 |
| 5 | 514 | 0 | 514 | 0.0511 | 0.0082 | 0.0372 | 0.0699 |
| 2012 | | | | | | | |
| .0794 | 785 | 6 | 63 | 0.0189 | 0.0048 | 0.0114 | 0.0311 |
| 1 | 716 | 12 | 103 | 0.0267 | 0.0057 | 0.0175 | 0.0407 |
| 5 | 601 | 0 | 601 | 0.0427 | 0.0073 | 0.0306 | 0.0596 |
| 2013 | | | | | | | |
| .0794 | 773 | 8 | -12 | 0.0313 | 0.0062 | 0.0213 | 0.0460 |
| 1 | 777 | 6 | 68 | 0.0414 | 0.0071 | 0.0296 | 0.0577 |
| 5 | 703 | 0 | 703 | 0.0494 | 0.0077 | 0.0364 | 0.0670 |
| 2014 | | | | | | | |
| .0794 | 741 | 8 | -32 | 0.0193 | 0.0049 | 0.0117 | 0.0318 |
| 1 | 765 | 7 | 85 | 0.0297 | 0.0061 | 0.0199 | 0.0444 |
| 5 | 673 | 0 | 673 | 0.0386 | 0.0069 | 0.0271 | 0.0547 |
| 2015 | | | | | | | |
| .0794 | 748 | 7 | -11 | 0.0635 | 0.0082 | 0.0492 | 0.0817 |
| 1 | 752 | 15 | -3 | 0.0721 | 0.0088 | 0.0567 | 0.0913 |
| 5 | 740 | 0 | 740 | 0.0907 | 0.0098 | 0.0733 | 0.1120 |

```
------------------------------------------------------------------------
Notes: Survival-time summaries shown for specified times in (0, 5].
       Net lost equals the number lost minus the number who entered.




. sts list if year>10 & site=="FFN" & survey==1, by(year) failure
risktable(.0793977 1 5)

         Failure _d: endevent==1
   Analysis time _t: (enddate-origin)/365.25
             Origin: time dob
  Enter on or after: time startdate
        ID variable: id

Kaplan–Meier failure function
By variable: year

             At           Net     Failure      Std.
  Time     risk   Fail   lost    function     error     [95% conf. int.]
------------------------------------------------------------------------
2001
 .0794      202     10     -1      0.0315    0.0126     0.0143    0.0687
     1      193     13     -5      0.0808    0.0194     0.0503    0.1286
     5      185      0    185      0.1453    0.0250     0.1032    0.2025
2002
 .0794      304      7   -137      0.0300    0.0099     0.0157    0.0569
     1      434     23     34      0.0479    0.0118     0.0295    0.0774
     5      377      0    377      0.1000    0.0154     0.0738    0.1348
2003
 .0794      615     12     41      0.0143    0.0047     0.0074    0.0272
     1      562     26   -100      0.0346    0.0074     0.0227    0.0525
     5      636      0    636      0.0727    0.0102     0.0551    0.0956
2004
 .0794      681     15    -13      0.0043    0.0025     0.0014    0.0134
     1      679     25     11      0.0259    0.0060     0.0164    0.0407
     5      643      0    643      0.0631    0.0093     0.0471    0.0841
2005
 .0794      708      9     -5      0.0239    0.0057     0.0149    0.0381
     1      704     15     20      0.0362    0.0070     0.0248    0.0527
     5      669      0    669      0.0581    0.0088     0.0431    0.0781
2006
 .0794      721      9    -14      0.0107    0.0038     0.0054    0.0214
     1      726     15     38      0.0233    0.0056     0.0146    0.0372
     5      673      0    673      0.0442    0.0076     0.0314    0.0619
2007
 .0794      816     11     40      0.0240    0.0053     0.0155    0.0369
     1      765     14    144      0.0373    0.0066     0.0264    0.0526
     5      607      0    607      0.0558    0.0081     0.0419    0.0740
2008
 .0794      846     12      7      0.0107    0.0035     0.0056    0.0204
     1      827     13     89      0.0249    0.0054     0.0163    0.0379
     5      725      0    725      0.0412    0.0069     0.0296    0.0573
```

```
2009
 .0794       736      10   -115       0.0000         .           .          .
     1       841      16     79       0.0122    0.0038      0.0066     0.0226
     5       746       0    746       0.0311    0.0060      0.0213     0.0453
2010
 .0794       845       7     25       0.0037    0.0021      0.0012     0.0114
     1       813      17      9       0.0118    0.0037      0.0063     0.0218
     5       787       0    787       0.0319    0.0061      0.0220     0.0462
2011
 .0794       854       6    -10       0.0114    0.0036      0.0061     0.0211
     1       858      18     19       0.0183    0.0045      0.0113     0.0298
     5       821       0    821       0.0384    0.0065      0.0276     0.0534
2012
 .0794      1053      14    149       0.0177    0.0040      0.0114     0.0277
     1       890      14     -6       0.0317    0.0054      0.0226     0.0443
     5       882       0    882       0.0466    0.0067      0.0352     0.0616
2013
 .0794      1004      11    -73       0.0130    0.0036      0.0076     0.0223
     1      1066      21    114       0.0234    0.0047      0.0158     0.0347
     5       931       0    931       0.0445    0.0065      0.0334     0.0591
2014
 .0794       911      12   -109       0.0158    0.0040      0.0095     0.0260
     1      1008      15     66       0.0281    0.0053      0.0193     0.0407
     5       927       0    927       0.0425    0.0064      0.0316     0.0571
2015
 .0794       867      11    -97       0.0107    0.0036      0.0056     0.0205
     1       953      15      5       0.0222    0.0049      0.0144     0.0342
     5       933       0    933       0.0369    0.0061      0.0266     0.0511
-----------------------------------------------------------------------
Notes: Survival-time summaries shown for specified times in (0, 5].
       Net lost equals the number lost minus the number who entered.
```

```
. sts list if year>10 & site=="BAS" & survey==0, by(year) failure
risktable(.0793977 1 5)

         Failure _d: endevent==1
   Analysis time _t: (enddate-origin)/365.25
             Origin: time dob
  Enter on or after: time startdate
        ID variable: id

Kaplan–Meier failure function
By variable: year

             At            Net     Failure      Std.
  Time     risk    Fail   lost    function     error     [95% conf. int.]
-----------------------------------------------------------------------
2001
 .0794       351      10    -53       0.0173    0.0070      0.0078     0.0382
     1       394      16    152       0.0438    0.0107      0.0270     0.0705
     5       226       0    226       0.0936    0.0160      0.0668     0.1304
```

| | | | | | | | |
|---|---|---|---|---|---|---|---|
| 2002 | | | | | | | |
| .0794 | 355 | 13 | 9 | 0.0403 | 0.0102 | 0.0245 | 0.0660 |
| 1 | 333 | 10 | 74 | 0.0762 | 0.0138 | 0.0532 | 0.1084 |
| 5 | 249 | 0 | 249 | 0.1030 | 0.0159 | 0.0760 | 0.1389 |
| 2003 | | | | | | | |
| .0794 | 407 | 13 | 52 | 0.0325 | 0.0086 | 0.0194 | 0.0543 |
| 1 | 342 | 14 | 47 | 0.0658 | 0.0123 | 0.0455 | 0.0945 |
| 5 | 281 | 0 | 281 | 0.1035 | 0.0154 | 0.0771 | 0.1382 |
| 2004 | | | | | | | |
| .0794 | 411 | 7 | -4 | 0.0352 | 0.0089 | 0.0214 | 0.0578 |
| 1 | 408 | 18 | 123 | 0.0514 | 0.0107 | 0.0342 | 0.0771 |
| 5 | 267 | 0 | 267 | 0.1002 | 0.0151 | 0.0743 | 0.1343 |
| 2005 | | | | | | | |
| .0794 | 494 | 12 | 81 | 0.0195 | 0.0061 | 0.0106 | 0.0360 |
| 1 | 401 | 14 | 15 | 0.0452 | 0.0094 | 0.0299 | 0.0679 |
| 5 | 372 | 0 | 372 | 0.0805 | 0.0130 | 0.0586 | 0.1102 |
| 2006 | | | | | | | |
| .0794 | 480 | 15 | -21 | 0.0227 | 0.0068 | 0.0126 | 0.0406 |
| 1 | 486 | 12 | 154 | 0.0531 | 0.0101 | 0.0365 | 0.0770 |
| 5 | 320 | 0 | 320 | 0.0801 | 0.0125 | 0.0589 | 0.1086 |
| 2007 | | | | | | | |
| .0794 | 556 | 8 | 83 | 0.0245 | 0.0065 | 0.0146 | 0.0410 |
| 1 | 465 | 10 | 127 | 0.0396 | 0.0083 | 0.0262 | 0.0596 |
| 5 | 328 | 0 | 328 | 0.0621 | 0.0107 | 0.0442 | 0.0870 |
| 2008 | | | | | | | |
| .0794 | 574 | 6 | 20 | 0.0269 | 0.0066 | 0.0165 | 0.0435 |
| 1 | 548 | 12 | 139 | 0.0371 | 0.0078 | 0.0246 | 0.0559 |
| 5 | 397 | 0 | 397 | 0.0608 | 0.0102 | 0.0437 | 0.0842 |
| 2009 | | | | | | | |
| .0794 | 636 | 9 | 51 | 0.0108 | 0.0041 | 0.0052 | 0.0225 |
| 1 | 576 | 9 | 179 | 0.0253 | 0.0062 | 0.0156 | 0.0410 |
| 5 | 388 | 0 | 388 | 0.0423 | 0.0083 | 0.0287 | 0.0621 |
| 2010 | | | | | | | |
| .0794 | 670 | 14 | 21 | 0.0089 | 0.0036 | 0.0040 | 0.0198 |
| 1 | 635 | 16 | 146 | 0.0315 | 0.0068 | 0.0206 | 0.0479 |
| 5 | 473 | 0 | 473 | 0.0581 | 0.0095 | 0.0421 | 0.0798 |
| 2011 | | | | | | | |
| .0794 | 669 | 6 | 10 | 0.0118 | 0.0041 | 0.0059 | 0.0235 |
| 1 | 653 | 13 | 184 | 0.0207 | 0.0055 | 0.0123 | 0.0347 |
| 5 | 456 | 0 | 456 | 0.0426 | 0.0081 | 0.0294 | 0.0617 |
| 2012 | | | | | | | |
| .0794 | 735 | 8 | 67 | 0.0146 | 0.0044 | 0.0081 | 0.0263 |
| 1 | 660 | 10 | 116 | 0.0275 | 0.0061 | 0.0178 | 0.0423 |
| 5 | 534 | 0 | 534 | 0.0410 | 0.0075 | 0.0287 | 0.0585 |
| 2013 | | | | | | | |
| .0794 | 694 | 7 | -50 | 0.0194 | 0.0051 | 0.0115 | 0.0326 |
| 1 | 737 | 20 | 155 | 0.0290 | 0.0062 | 0.0190 | 0.0442 |
| 5 | 562 | 0 | 562 | 0.0580 | 0.0088 | 0.0430 | 0.0780 |
| 2014 | | | | | | | |
| .0794 | 722 | 8 | 6 | 0.0271 | 0.0060 | 0.0176 | 0.0417 |
| 1 | 708 | 10 | 79 | 0.0383 | 0.0071 | 0.0266 | 0.0550 |
| 5 | 619 | 0 | 619 | 0.0521 | 0.0082 | 0.0382 | 0.0709 |
| 2015 | | | | | | | |
| .0794 | 720 | 10 | 8 | 0.0163 | 0.0047 | 0.0093 | 0.0286 |
| 1 | 702 | 21 | 39 | 0.0298 | 0.0063 | 0.0197 | 0.0449 |

```
     5      642      0    642      0.0590    0.0087     0.0441    0.0788
-------------------------------------------------------------------------
Notes: Survival-time summaries shown for specified times in (0, 5].
       Net lost equals the number lost minus the number who entered.
```

```
. sts list if year>10 & site=="BAS" & survey==1, by(year) failure
risktable(.0793977 1 5)

         Failure _d: endevent==1
   Analysis time _t: (enddate-origin)/365.25
             Origin: time dob
  Enter on or after: time startdate
        ID variable: id

Kaplan–Meier failure function
By variable: year

             At           Net     Failure      Std.
  Time     risk   Fail   lost    function     error     [95% conf. int.]
-------------------------------------------------------------------------
2006
 .0794      543     10   -340      0.0074    0.0037     0.0028    0.0196
     1      873     25     95      0.0205    0.0056     0.0120    0.0348
     5      753      0    753      0.0523    0.0083     0.0383    0.0713
2007
 .0794     1470     16    236      0.0048    0.0018     0.0023    0.0100
     1     1218     50    -42      0.0172    0.0036     0.0115    0.0259
     5     1210      0   1210      0.0539    0.0062     0.0431    0.0674
2008
 .0794     1475     25   -200      0.0101    0.0026     0.0061    0.0168
     1     1650     48    261      0.0261    0.0041     0.0192    0.0354
     5     1341      0   1341      0.0584    0.0060     0.0476    0.0715
2009
 .0794     1659     39      6      0.0177    0.0032     0.0124    0.0252
     1     1614     52    331      0.0404    0.0048     0.0320    0.0508
     5     1231      0   1231      0.0709    0.0062     0.0596    0.0841
2010
 .0794     1826     40    -10      0.0116    0.0025     0.0076    0.0177
     1     1796     82     18      0.0333    0.0042     0.0260    0.0426
     5     1696      0   1696      0.0798    0.0064     0.0682    0.0934
2011
 .0794     1820     37   -134      0.0165    0.0030     0.0115    0.0235
     1     1917     59    406      0.0358    0.0043     0.0283    0.0453
     5     1452      0   1452      0.0674    0.0058     0.0569    0.0798
2012
 .0794     1938     34     36      0.0146    0.0027     0.0102    0.0210
     1     1868     57     28      0.0319    0.0040     0.0250    0.0406
     5     1783      0   1783      0.0615    0.0054     0.0517    0.0731
2013
 .0794     2011     38     -5      0.0140    0.0026     0.0097    0.0202
     1     1978     59    294      0.0324    0.0039     0.0256    0.0411
     5     1625      0   1625      0.0624    0.0054     0.0526    0.0739
```

```
2014
 .0794     1879      25   -123      0.0187    0.0031     0.0135    0.0258
     1     1977      48    147      0.0316    0.0040     0.0247    0.0405
     5     1782       0   1782      0.0553    0.0052     0.0461    0.0664
2015
 .0794     1662      24   -262      0.0178    0.0033     0.0124    0.0255
     1     1900      36     -3      0.0304    0.0041     0.0233    0.0397
     5     1867       0   1867      0.0487    0.0051     0.0397    0.0597
------------------------------------------------------------------------
Notes: Survival-time summaries shown for specified times in (0, 5].
       Net lost equals the number lost minus the number who entered.
```

CHILD MORTALITY

```
. sts list if year>10 & site=="FFN" & survey==0, by(per) failure risktable(4)

         Failure _d: endevent==1
   Analysis time _t: (enddate-origin)/365.25
             Origin: time (dob+365.25)
  Enter on or after: time startdate
        ID variable: id
              Notes: age>4 trimmed
                     year>26 trimmed

Kaplan–Meier failure function
By variable: per

             At           Net     Failure      Std.
  Time     risk   Fail   lost    function     error     [95% conf. int.]
------------------------------------------------------------------------
2001-2005
     4     1591       0   1591      0.0301    0.0039     0.0233    0.0389
2006-2010
     4     2173       0   2173      0.0131    0.0022     0.0094    0.0181
2011-2015
     4     3231       0   3231      0.0146    0.0020     0.0111    0.0190
------------------------------------------------------------------------
Notes: Survival-time summaries shown for specified times in (0, 4].
       Net lost equals the number lost minus the number who entered.

. sts list if year>10 & site=="FFN" & survey==1, by(per) failure risktable(4)

         Failure _d: endevent==1
   Analysis time _t: (enddate-origin)/365.25
             Origin: time (dob+365.25)
  Enter on or after: time startdate
        ID variable: id
              Notes: age>4 trimmed
                     year>26 trimmed
```

```
Kaplan–Meier failure function
By variable: per

             At           Net     Failure      Std.
  Time     risk   Fail   lost    function     error     [95% conf. int.]
------------------------------------------------------------------------
2001-2005
     4     2511      0   2511      0.0392    0.0038     0.0324    0.0474
2006-2010
     4     3538      0   3538      0.0192    0.0022     0.0153    0.0240
2011-2015
     4     4497      0   4497      0.0174    0.0019     0.0141    0.0216
------------------------------------------------------------------------
Notes: Survival-time summaries shown for specified times in (0, 4].
       Net lost equals the number lost minus the number who entered.

. sts list if year>10 & site=="BAS" & survey==0, by(per) failure risktable(4)

         Failure _d: endevent==1
   Analysis time _t: (enddate-origin)/365.25
             Origin: time (dob+365.25)
  Enter on or after: time startdate
        ID variable: id
              Notes: age>4 trimmed
                     year>26 trimmed

Kaplan–Meier failure function
By variable: per

             At           Net     Failure      Std.
  Time     risk   Fail   lost    function     error     [95% conf. int.]
------------------------------------------------------------------------
2001-2005
     4     1395      0   1395      0.0425    0.0049     0.0338    0.0532
2006-2010
     4     1906      0   1906      0.0242    0.0032     0.0187    0.0312
2011-2015
     4     2813      0   2813      0.0223    0.0026     0.0177    0.0279
------------------------------------------------------------------------
Notes: Survival-time summaries shown for specified times in (0, 4].
       Net lost equals the number lost minus the number who entered.

. sts list if year>10 & site=="BAS" & survey==1, by(per) failure risktable(4)

         Failure _d: endevent==1
   Analysis time _t: (enddate-origin)/365.25
             Origin: time (dob+365.25)
  Enter on or after: time startdate
        ID variable: id
              Notes: age>4 trimmed
                     year>26 trimmed

Kaplan–Meier failure function
By variable: per
```

```
             At           Net     Failure      Std.
  Time     risk   Fail   lost    function     error     [95% conf. int.]
------------------------------------------------------------------------
2001-2005
     4        4      0      4      0.0000         .          .         .
2006-2010
     4     6231      0   6231      0.0373    0.0023     0.0331    0.0421
2011-2015
     4     8509      0   8509      0.0274    0.0017     0.0243    0.0309
------------------------------------------------------------------------
Notes: Survival-time summaries shown for specified times in (0, 4].
       Net lost equals the number lost minus the number who entered.

.
.
. sts list if year>10 & site=="FFN" & survey==0, by(year) failure risktable(4)

         Failure _d: endevent==1
   Analysis time _t: (enddate-origin)/365.25
             Origin: time (dob+365.25)
  Enter on or after: time startdate
        ID variable: id
              Notes: age>4 trimmed
                     year>26 trimmed

Kaplan-Meier failure function
By variable: year

             At           Net     Failure      Std.
  Time     risk   Fail   lost    function     error     [95% conf. int.]
------------------------------------------------------------------------
2001
     4      232      0    232      0.0335    0.0105     0.0181    0.0617
2002
     4      287      0    287      0.0356    0.0096     0.0209    0.0601
2003
     4      296      0    296      0.0289    0.0086     0.0160    0.0518
2004
     4      321      0    321      0.0349    0.0092     0.0208    0.0584
2005
     4      455      0    455      0.0181    0.0064     0.0091    0.0359
2006
     4      348      0    348      0.0022    0.0022     0.0003    0.0155
2007
     4      376      0    376      0.0146    0.0056     0.0069    0.0307
2008
     4      433      0    433      0.0155    0.0052     0.0081    0.0297
2009
     4      454      0    454      0.0156    0.0049     0.0084    0.0289
2010
     4      562      0    562      0.0144    0.0048     0.0075    0.0275
2011
     4      514      0    514      0.0195    0.0054     0.0113    0.0335
2012
     4      601      0    601      0.0165    0.0047     0.0094    0.0288
```

```
2013
     4      703      0    703      0.0084    0.0034     0.0038    0.0186
2014
     4      673      0    673      0.0091    0.0034     0.0044    0.0190
2015
     4      740      0    740      0.0201    0.0051     0.0122    0.0331
-----------------------------------------------------------------------
Notes: Survival-time summaries shown for specified times in (0, 4].
       Net lost equals the number lost minus the number who entered.

. sts list if year>10 & site=="FFN" & survey==1, by(year) failure risktable(4)

         Failure _d: endevent==1
   Analysis time _t: (enddate-origin)/365.25
             Origin: time (dob+365.25)
  Enter on or after: time startdate
        ID variable: id
              Notes: age>4 trimmed
                     year>26 trimmed

Kaplan-Meier failure function
By variable: year

             At           Net     Failure      Std.
  Time     risk   Fail   lost    function     error     [95% conf. int.]
-----------------------------------------------------------------------
2001
     4      185      0    185      0.0702    0.0189     0.0412    0.1183
2002
     4      377      0    377      0.0548    0.0111     0.0367    0.0813
2003
     4      636      0    636      0.0395    0.0076     0.0270    0.0575
2004
     4      643      0    643      0.0382    0.0075     0.0260    0.0560
2005
     4      670      0    670      0.0228    0.0058     0.0138    0.0375
2006
     4      673      0    673      0.0213    0.0055     0.0129    0.0352
2007
     4      607      0    607      0.0192    0.0051     0.0114    0.0322
2008
     4      725      0    725      0.0167    0.0046     0.0098    0.0287
2009
     4      746      0    746      0.0191    0.0047     0.0117    0.0309
2010
     4      787      0    787      0.0204    0.0049     0.0127    0.0326
2011
     4      822      0    822      0.0204    0.0048     0.0129    0.0323
2012
     4      884      0    884      0.0154    0.0041     0.0091    0.0258
2013
     4      931      0    931      0.0216    0.0047     0.0141    0.0329
2014
     4      927      0    927      0.0149    0.0038     0.0090    0.0245
2015
```

```
     4      933      0    933      0.0151    0.0039     0.0091    0.0249
-----------------------------------------------------------------------
Notes: Survival-time summaries shown for specified times in (0, 4].
       Net lost equals the number lost minus the number who entered.

. sts list if year>10 & site=="BAS" & survey==0, by(year) failure risktable(4)

         Failure _d: endevent==1
   Analysis time _t: (enddate-origin)/365.25
             Origin: time (dob+365.25)
  Enter on or after: time startdate
        ID variable: id
              Notes: age>4 trimmed
                     year>26 trimmed

Kaplan–Meier failure function
By variable: year

             At           Net     Failure      Std.
  Time     risk   Fail   lost    function     error     [95% conf. int.]
-----------------------------------------------------------------------
2001
     4      226      0    226      0.0522    0.0129     0.0320    0.0845
2002
     4      249      0    249      0.0290    0.0091     0.0157    0.0535
2003
     4      281      0    281      0.0404    0.0106     0.0241    0.0674
2004
     4      267      0    267      0.0514    0.0118     0.0326    0.0805
2005
     4      372      0    372      0.0371    0.0098     0.0221    0.0619
2006
     4      320      0    320      0.0285    0.0082     0.0162    0.0498
2007
     4      328      0    328      0.0234    0.0073     0.0127    0.0432
2008
     4      397      0    397      0.0246    0.0070     0.0140    0.0430
2009
     4      388      0    388      0.0175    0.0058     0.0091    0.0334
2010
     4      473      0    473      0.0274    0.0070     0.0166    0.0452
2011
     4      456      0    456      0.0224    0.0062     0.0130    0.0383
2012
     4      534      0    534      0.0139    0.0046     0.0073    0.0266
2013
     4      562      0    562      0.0298    0.0066     0.0193    0.0459
2014
     4      619      0    619      0.0143    0.0045     0.0077    0.0264
2015
     4      642      0    642      0.0301    0.0065     0.0197    0.0458
-----------------------------------------------------------------------
Notes: Survival-time summaries shown for specified times in (0, 4].
       Net lost equals the number lost minus the number who entered.
```

```
. sts list if year>10 & site=="BAS" & survey==1, by(year) failure risktable(4)

         Failure _d: endevent==1
   Analysis time _t: (enddate-origin)/365.25
             Origin: time (dob+365.25)
  Enter on or after: time startdate
        ID variable: id
              Notes: age>4 trimmed
                     year>26 trimmed

Kaplan-Meier failure function
By variable: year

             At           Net     Failure      Std.
  Time     risk   Fail   lost    function     error     [95% conf. int.]
------------------------------------------------------------------------
2006
     4      753      0    753      0.0325    0.0064     0.0220    0.0479
2007
     4     1210      0   1210      0.0373    0.0052     0.0284    0.0490
2008
     4     1341      0   1341      0.0332    0.0047     0.0251    0.0438
2009
     4     1231      0   1231      0.0318    0.0044     0.0243    0.0416
2010
     4     1696      0   1696      0.0480    0.0052     0.0389    0.0593
2011
     4     1452      0   1452      0.0328    0.0042     0.0255    0.0421
2012
     4     1783      0   1783      0.0306    0.0040     0.0237    0.0395
2013
     4     1625      0   1625      0.0310    0.0040     0.0241    0.0399
2014
     4     1782      0   1782      0.0245    0.0035     0.0185    0.0323
2015
     4     1867      0   1867      0.0189    0.0031     0.0136    0.0260
------------------------------------------------------------------------
Notes: Survival-time summaries shown for specified times in (0, 4].
       Net lost equals the number lost minus the number who entered.



. log off
      name:  <unnamed>
       log:  C:\Users\mjasseh\......\Final Version\PLoS ONE Re-submission\Response
to Queries\Kaplan-Meier List.smcl
  log type:  smcl
 paused on:  19 Apr 2022, 16:22:29
--------------------------------------------------------------------------------
```
