# Supplementary material for: Assessment of the consistency of health and demographic surveillance and household survey data: A demonstration at two HDSS sites in The Gambia
Source: PLoS One. 2022 Jul 13;17(7):e0271464. doi: 10.1371/journal.pone.0271464 (PMC9278757; doi:10.1371/journal.pone.0271464)
Supplement: S4 File — (PDF) [file pone.0271464.s004.pdf]

RATE RATIO ESTIMATES

```
---------------------------------------------------------------------------
      name:  <unnamed>
       log:  C:\Users\mjasseh\......\Childhood Mortality Indicators by
Period - All - Stata 17.smcl
  log type:  smcl
 opened on:   5 May 2022, 16:12:28

.

. *** NEONATAL, INFANT & UNDER-5 *******************
.
. **FARAFENNI
. keep if site=="FFN"
.
. * PERIODIC COMPARISONS

. stmh survey if age==0 & per==3

         Failure _d: endevent==1
   Analysis time _t: (enddate-origin)/365.25
             Origin: time dob
  Enter on or after: time startdate
        ID variable: id
              Notes: age>5 trimmed
                     year>26 trimmed

Mantel–Haenszel estimate of the rate ratio
  comparing survey==1 vs. survey==0

-------------------------------------------------------------------------
    Rate ratio         chi2         P>chi2         [95% conf. interval]
-------------------------------------------------------------------------
         1.615         6.11         0.0134             1.100      2.371
-------------------------------------------------------------------------

. stmh survey if age<1 & per==3

         Failure _d: endevent==1
   Analysis time _t: (enddate-origin)/365.25
             Origin: time dob
  Enter on or after: time startdate
        ID variable: id
              Notes: age>5 trimmed
                     year>26 trimmed

Mantel–Haenszel estimate of the rate ratio
  comparing survey==1 vs. survey==0

-------------------------------------------------------------------------
```

```
    Rate ratio         chi2         P>chi2          [95% conf. interval]
-------------------------------------------------------------------------
         1.232         2.18         0.1396              0.934      1.626
-------------------------------------------------------------------------

. stmh survey if age<5 & per==3

         Failure _d: endevent==1
   Analysis time _t: (enddate-origin)/365.25
             Origin: time dob
  Enter on or after: time startdate
        ID variable: id
              Notes: age>5 trimmed
                     year>26 trimmed

Mantel–Haenszel estimate of the rate ratio
  comparing survey==1 vs. survey==0

-------------------------------------------------------------------------
    Rate ratio         chi2         P>chi2          [95% conf. interval]
-------------------------------------------------------------------------
         1.077         0.49         0.4843              0.875      1.326
-------------------------------------------------------------------------

.
. stmh survey if age==0 & per==4

         Failure _d: endevent==1
   Analysis time _t: (enddate-origin)/365.25
             Origin: time dob
  Enter on or after: time startdate
        ID variable: id
              Notes: age>5 trimmed
                     year>26 trimmed

Mantel–Haenszel estimate of the rate ratio
  comparing survey==1 vs. survey==0

-------------------------------------------------------------------------
    Rate ratio         chi2         P>chi2          [95% conf. interval]
-------------------------------------------------------------------------
         2.695        29.53         0.0000              1.857      3.912
-------------------------------------------------------------------------

. stmh survey if age<1 & per==4

         Failure _d: endevent==1
   Analysis time _t: (enddate-origin)/365.25
             Origin: time dob
  Enter on or after: time startdate
```

```
        ID variable: id
              Notes: age>5 trimmed
                     year>26 trimmed

Mantel-Haenszel estimate of the rate ratio
  comparing survey==1 vs. survey==0

-------------------------------------------------------------------------
    Rate ratio         chi2         P>chi2          [95% conf. interval]
-------------------------------------------------------------------------
         1.710        15.22         0.0001             1.302      2.246
-------------------------------------------------------------------------

. stmh survey if age<5 & per==4

         Failure _d: endevent==1
   Analysis time _t: (enddate-origin)/365.25
             Origin: time dob
  Enter on or after: time startdate
        ID variable: id
              Notes: age>5 trimmed
                     year>26 trimmed

Mantel-Haenszel estimate of the rate ratio
  comparing survey==1 vs. survey==0

-------------------------------------------------------------------------
    Rate ratio         chi2         P>chi2          [95% conf. interval]
-------------------------------------------------------------------------
         1.365         7.87         0.0050             1.097      1.697
-------------------------------------------------------------------------

.
. stmh survey if age==0 & per==5

         Failure _d: endevent==1
   Analysis time _t: (enddate-origin)/365.25
             Origin: time dob
  Enter on or after: time startdate
        ID variable: id
              Notes: age>5 trimmed
                     year>26 trimmed

Mantel-Haenszel estimate of the rate ratio
  comparing survey==1 vs. survey==0

-------------------------------------------------------------------------
    Rate ratio         chi2         P>chi2          [95% conf. interval]
-------------------------------------------------------------------------
         2.361        34.07         0.0000             1.754      3.179
```

```
------------------------------------------------------------------------

. stmh survey if age<1 & per==5

         Failure _d: endevent==1
   Analysis time _t: (enddate-origin)/365.25
             Origin: time dob
  Enter on or after: time startdate
        ID variable: id
              Notes: age>5 trimmed
                     year>26 trimmed

Mantel-Haenszel estimate of the rate ratio
  comparing survey==1 vs. survey==0

------------------------------------------------------------------------
    Rate ratio         chi2         P>chi2         [95% conf. interval]
------------------------------------------------------------------------
         1.727        21.20         0.0000             1.365       2.185
------------------------------------------------------------------------

. stmh survey if age<5 & per==5

         Failure _d: endevent==1
   Analysis time _t: (enddate-origin)/365.25
             Origin: time dob
  Enter on or after: time startdate
        ID variable: id
              Notes: age>5 trimmed
                     year>26 trimmed

Mantel-Haenszel estimate of the rate ratio
  comparing survey==1 vs. survey==0

------------------------------------------------------------------------
    Rate ratio         chi2         P>chi2         [95% conf. interval]
------------------------------------------------------------------------
         1.402        12.10         0.0005             1.158       1.698
------------------------------------------------------------------------

.
.
. * ANNUAL COMPARISONS
. stmh survey if age==0 & year==21

         Failure _d: endevent==1
   Analysis time _t: (enddate-origin)/365.25
             Origin: time dob
  Enter on or after: time startdate
        ID variable: id
```

```
             Notes: age>5 trimmed
                    year>26 trimmed

Mantel–Haenszel estimate of the rate ratio
  comparing survey==1 vs. survey==0

------------------------------------------------------------------------
    Rate ratio         chi2         P>chi2          [95% conf. interval]
------------------------------------------------------------------------
         1.905         2.65         0.1038             0.865      4.198
------------------------------------------------------------------------

. stmh survey if age<1 & year==21

         Failure _d: endevent==1
   Analysis time _t: (enddate-origin)/365.25
             Origin: time dob
  Enter on or after: time startdate
        ID variable: id
             Notes: age>5 trimmed
                    year>26 trimmed

Mantel–Haenszel estimate of the rate ratio
  comparing survey==1 vs. survey==0

------------------------------------------------------------------------
    Rate ratio         chi2         P>chi2          [95% conf. interval]
------------------------------------------------------------------------
         1.743         3.04         0.0811             0.926      3.282
------------------------------------------------------------------------

. stmh survey if age<5 & year==21

         Failure _d: endevent==1
   Analysis time _t: (enddate-origin)/365.25
             Origin: time dob
  Enter on or after: time startdate
        ID variable: id
             Notes: age>5 trimmed
                    year>26 trimmed

Mantel–Haenszel estimate of the rate ratio
  comparing survey==1 vs. survey==0

------------------------------------------------------------------------
    Rate ratio         chi2         P>chi2          [95% conf. interval]
------------------------------------------------------------------------
         1.389         1.93         0.1645             0.872      2.213
------------------------------------------------------------------------
```

```
.
. stmh survey if age==0 & year==22

         Failure _d: endevent==1
   Analysis time _t: (enddate-origin)/365.25
             Origin: time dob
  Enter on or after: time startdate
        ID variable: id
              Notes: age>5 trimmed
                     year>26 trimmed

Mantel-Haenszel estimate of the rate ratio
  comparing survey==1 vs. survey==0

------------------------------------------------------------------------
    Rate ratio         chi2         P>chi2          [95% conf. interval]
------------------------------------------------------------------------
         1.062         0.03         0.8620              0.540      2.090
------------------------------------------------------------------------

. stmh survey if age<1 & year==22

         Failure _d: endevent==1
   Analysis time _t: (enddate-origin)/365.25
             Origin: time dob
  Enter on or after: time startdate
        ID variable: id
              Notes: age>5 trimmed
                     year>26 trimmed

Mantel-Haenszel estimate of the rate ratio
  comparing survey==1 vs. survey==0

------------------------------------------------------------------------
    Rate ratio         chi2         P>chi2          [95% conf. interval]
------------------------------------------------------------------------
         0.833         0.43         0.5112              0.482      1.439
------------------------------------------------------------------------

. stmh survey if age<5 & year==22

         Failure _d: endevent==1
   Analysis time _t: (enddate-origin)/365.25
             Origin: time dob
  Enter on or after: time startdate
        ID variable: id
              Notes: age>5 trimmed
                     year>26 trimmed

Mantel-Haenszel estimate of the rate ratio
```

```
  comparing survey==1 vs. survey==0

------------------------------------------------------------------------
    Rate ratio         chi2         P>chi2          [95% conf. interval]
------------------------------------------------------------------------
         0.910         0.17         0.6765              0.583      1.420
------------------------------------------------------------------------

.
. stmh survey if age==0 & year==23

         Failure _d: endevent==1
   Analysis time _t: (enddate-origin)/365.25
             Origin: time dob
  Enter on or after: time startdate
        ID variable: id
              Notes: age>5 trimmed
                     year>26 trimmed

Mantel–Haenszel estimate of the rate ratio
  comparing survey==1 vs. survey==0

------------------------------------------------------------------------
    Rate ratio         chi2         P>chi2          [95% conf. interval]
------------------------------------------------------------------------
         2.473         7.50         0.0062              1.265      4.834
------------------------------------------------------------------------

. stmh survey if age<1 & year==23

         Failure _d: endevent==1
   Analysis time _t: (enddate-origin)/365.25
             Origin: time dob
  Enter on or after: time startdate
        ID variable: id
              Notes: age>5 trimmed
                     year>26 trimmed

Mantel–Haenszel estimate of the rate ratio
  comparing survey==1 vs. survey==0

------------------------------------------------------------------------
    Rate ratio         chi2         P>chi2          [95% conf. interval]
------------------------------------------------------------------------
         1.851         5.43         0.0198              1.094      3.131
------------------------------------------------------------------------

. stmh survey if age<5 & year==23

         Failure _d: endevent==1
```

```
   Analysis time _t: (enddate-origin)/365.25
             Origin: time dob
  Enter on or after: time startdate
        ID variable: id
              Notes: age>5 trimmed
                     year>26 trimmed

Mantel-Haenszel estimate of the rate ratio
  comparing survey==1 vs. survey==0

------------------------------------------------------------------------
    Rate ratio         chi2         P>chi2          [95% conf. interval]
------------------------------------------------------------------------
         1.143         0.37         0.5403             0.745      1.755
------------------------------------------------------------------------

.
. stmh survey if age==0 & year==24

         Failure _d: endevent==1
   Analysis time _t: (enddate-origin)/365.25
             Origin: time dob
  Enter on or after: time startdate
        ID variable: id
              Notes: age>5 trimmed
                     year>26 trimmed

Mantel-Haenszel estimate of the rate ratio
  comparing survey==1 vs. survey==0

------------------------------------------------------------------------
    Rate ratio         chi2         P>chi2          [95% conf. interval]
------------------------------------------------------------------------
         1.227         0.32         0.5741             0.600      2.511
------------------------------------------------------------------------

. stmh survey if age<1 & year==24

         Failure _d: endevent==1
   Analysis time _t: (enddate-origin)/365.25
             Origin: time dob
  Enter on or after: time startdate
        ID variable: id
              Notes: age>5 trimmed
                     year>26 trimmed

Mantel-Haenszel estimate of the rate ratio
  comparing survey==1 vs. survey==0

------------------------------------------------------------------------
```

```
    Rate ratio         chi2         P>chi2          [95% conf. interval]
------------------------------------------------------------------------
         1.090         0.09         0.7611             0.625      1.901
------------------------------------------------------------------------

. stmh survey if age<5 & year==24

         Failure _d: endevent==1
   Analysis time _t: (enddate-origin)/365.25
             Origin: time dob
  Enter on or after: time startdate
        ID variable: id
              Notes: age>5 trimmed
                     year>26 trimmed

Mantel–Haenszel estimate of the rate ratio
  comparing survey==1 vs. survey==0

------------------------------------------------------------------------
    Rate ratio         chi2         P>chi2          [95% conf. interval]
------------------------------------------------------------------------
         0.955         0.04         0.8473             0.598      1.526
------------------------------------------------------------------------

.
. stmh survey if age==0 & year==25

         Failure _d: endevent==1
   Analysis time _t: (enddate-origin)/365.25
             Origin: time dob
  Enter on or after: time startdate
        ID variable: id
              Notes: age>5 trimmed
                     year>26 trimmed

Mantel–Haenszel estimate of the rate ratio
  comparing survey==1 vs. survey==0

------------------------------------------------------------------------
    Rate ratio         chi2         P>chi2          [95% conf. interval]
------------------------------------------------------------------------
         6.743        37.92         0.0000             3.335     13.630
------------------------------------------------------------------------

. stmh survey if age<1 & year==25

         Failure _d: endevent==1
   Analysis time _t: (enddate-origin)/365.25
             Origin: time dob
  Enter on or after: time startdate
```

```
        ID variable: id
              Notes: age>5 trimmed
                     year>26 trimmed

Mantel–Haenszel estimate of the rate ratio
  comparing survey==1 vs. survey==0

------------------------------------------------------------------------
    Rate ratio         chi2         P>chi2          [95% conf. interval]
------------------------------------------------------------------------
         3.836        31.83         0.0000             2.320      6.344
------------------------------------------------------------------------

. stmh survey if age<5 & year==25

         Failure _d: endevent==1
   Analysis time _t: (enddate-origin)/365.25
             Origin: time dob
  Enter on or after: time startdate
        ID variable: id
              Notes: age>5 trimmed
                     year>26 trimmed

Mantel–Haenszel estimate of the rate ratio
  comparing survey==1 vs. survey==0

------------------------------------------------------------------------
    Rate ratio         chi2         P>chi2          [95% conf. interval]
------------------------------------------------------------------------
         2.957        31.28         0.0000             1.984      4.405
------------------------------------------------------------------------
```

```
. **BASSE
. keep if site=="BAS"
. 
. * PERIODIC COMPARISONS

. stmh survey if age==0 & per==4

         Failure _d: endevent==1
   Analysis time _t: (enddate-origin)/365.25
             Origin: time dob
  Enter on or after: time startdate
        ID variable: id
              Notes: age>5 trimmed
                     year>26 trimmed

Mantel–Haenszel estimate of the rate ratio
  comparing survey==1 vs. survey==0

-------------------------------------------------------------------------
    Rate ratio         chi2         P>chi2          [95% conf. interval]
-------------------------------------------------------------------------
         1.666         8.45         0.0036               1.177      2.359
-------------------------------------------------------------------------

. stmh survey if age<1 & per==4

         Failure _d: endevent==1
   Analysis time _t: (enddate-origin)/365.25
             Origin: time dob
  Enter on or after: time startdate
        ID variable: id
              Notes: age>5 trimmed
                     year>26 trimmed

Mantel–Haenszel estimate of the rate ratio
  comparing survey==1 vs. survey==0

-------------------------------------------------------------------------
    Rate ratio         chi2         P>chi2          [95% conf. interval]
-------------------------------------------------------------------------
         1.293         4.70         0.0302               1.024      1.633
-------------------------------------------------------------------------

. stmh survey if age<5 & per==4

         Failure _d: endevent==1
   Analysis time _t: (enddate-origin)/365.25
             Origin: time dob
  Enter on or after: time startdate
```

```
        ID variable: id
              Notes: age>5 trimmed
                     year>26 trimmed

Mantel–Haenszel estimate of the rate ratio
  comparing survey==1 vs. survey==0

------------------------------------------------------------------------
    Rate ratio         chi2         P>chi2          [95% conf. interval]
------------------------------------------------------------------------
         1.004         0.00         0.9626             0.841      1.199
------------------------------------------------------------------------

.
. stmh survey if age==0 & per==5

         Failure _d: endevent==1
   Analysis time _t: (enddate-origin)/365.25
             Origin: time dob
  Enter on or after: time startdate
        ID variable: id
              Notes: age>5 trimmed
                     year>26 trimmed

Mantel–Haenszel estimate of the rate ratio
  comparing survey==1 vs. survey==0

------------------------------------------------------------------------
    Rate ratio         chi2         P>chi2          [95% conf. interval]
------------------------------------------------------------------------
         1.114         0.53         0.4661             0.833      1.489
------------------------------------------------------------------------

. stmh survey if age<1 & per==5

         Failure _d: endevent==1
   Analysis time _t: (enddate-origin)/365.25
             Origin: time dob
  Enter on or after: time startdate
        ID variable: id
              Notes: age>5 trimmed
                     year>26 trimmed

Mantel–Haenszel estimate of the rate ratio
  comparing survey==1 vs. survey==0

------------------------------------------------------------------------
    Rate ratio         chi2         P>chi2          [95% conf. interval]
------------------------------------------------------------------------
         0.920         0.55         0.4583             0.737      1.147
```

```
------------------------------------------------------------------------

. stmh survey if age<5 & per==5

         Failure _d: endevent==1
   Analysis time _t: (enddate-origin)/365.25
             Origin: time dob
  Enter on or after: time startdate
        ID variable: id
              Notes: age>5 trimmed
                     year>26 trimmed

Mantel-Haenszel estimate of the rate ratio
  comparing survey==1 vs. survey==0

------------------------------------------------------------------------
    Rate ratio         chi2         P>chi2          [95% conf. interval]
------------------------------------------------------------------------
         0.893         1.75         0.1861              0.754      1.056
------------------------------------------------------------------------

.
.
. * ANNUAL COMPARISONS
stmh survey if age==0 & year==21

         Failure _d: endevent==1
   Analysis time _t: (enddate-origin)/365.25
             Origin: time dob
  Enter on or after: time startdate
        ID variable: id
              Notes: age>5 trimmed
                     year>26 trimmed

Mantel-Haenszel estimate of the rate ratio
  comparing survey==1 vs. survey==0

------------------------------------------------------------------------
    Rate ratio         chi2         P>chi2          [95% conf. interval]
------------------------------------------------------------------------
         0.717         0.70         0.4012              0.329      1.564
------------------------------------------------------------------------

. stmh survey if age<1 & year==21

         Failure _d: endevent==1
   Analysis time _t: (enddate-origin)/365.25
             Origin: time dob
  Enter on or after: time startdate
        ID variable: id
```

```
              Notes: age>5 trimmed
                     year>26 trimmed

Mantel-Haenszel estimate of the rate ratio
  comparing survey==1 vs. survey==0

------------------------------------------------------------------------
    Rate ratio         chi2         P>chi2          [95% conf. interval]
------------------------------------------------------------------------
         0.582         3.47         0.0623              0.327      1.035
------------------------------------------------------------------------

. stmh survey if age<5 & year==21

         Failure _d: endevent==1
   Analysis time _t: (enddate-origin)/365.25
             Origin: time dob
  Enter on or after: time startdate
        ID variable: id
              Notes: age>5 trimmed
                     year>26 trimmed

Mantel-Haenszel estimate of the rate ratio
  comparing survey==1 vs. survey==0

------------------------------------------------------------------------
    Rate ratio         chi2         P>chi2          [95% conf. interval]
------------------------------------------------------------------------
         0.642         4.43         0.0353              0.424      0.973
------------------------------------------------------------------------


.
. stmh survey if age==0 & year==22

         Failure _d: endevent==1
   Analysis time _t: (enddate-origin)/365.25
             Origin: time dob
  Enter on or after: time startdate
        ID variable: id
              Notes: age>5 trimmed
                     year>26 trimmed

Mantel-Haenszel estimate of the rate ratio
  comparing survey==1 vs. survey==0

------------------------------------------------------------------------
    Rate ratio         chi2         P>chi2          [95% conf. interval]
------------------------------------------------------------------------
         0.999         0.00         0.9975              0.499      2.000
------------------------------------------------------------------------
```

```
. stmh survey if age<1 & year==22

         Failure _d: endevent==1
   Analysis time _t: (enddate-origin)/365.25
             Origin: time dob
  Enter on or after: time startdate
        ID variable: id
              Notes: age>5 trimmed
                     year>26 trimmed

Mantel-Haenszel estimate of the rate ratio
  comparing survey==1 vs. survey==0

-------------------------------------------------------------------------
    Rate ratio         chi2         P>chi2          [95% conf. interval]
-------------------------------------------------------------------------
         0.875         0.27         0.6014                0.529      1.446
-------------------------------------------------------------------------

. stmh survey if age<5 & year==22

         Failure _d: endevent==1
   Analysis time _t: (enddate-origin)/365.25
             Origin: time dob
  Enter on or after: time startdate
        ID variable: id
              Notes: age>5 trimmed
                     year>26 trimmed

Mantel-Haenszel estimate of the rate ratio
  comparing survey==1 vs. survey==0

-------------------------------------------------------------------------
    Rate ratio         chi2         P>chi2          [95% conf. interval]
-------------------------------------------------------------------------
         0.707         2.83         0.0926                0.471      1.061
-------------------------------------------------------------------------


.
. stmh survey if age==0 & year==23

         Failure _d: endevent==1
   Analysis time _t: (enddate-origin)/365.25
             Origin: time dob
  Enter on or after: time startdate
        ID variable: id
              Notes: age>5 trimmed
                     year>26 trimmed
```

```
Mantel–Haenszel estimate of the rate ratio
  comparing survey==1 vs. survey==0

------------------------------------------------------------------------
    Rate ratio         chi2         P>chi2          [95% conf. interval]
------------------------------------------------------------------------
         1.417         1.15         0.2846              0.746      2.691
------------------------------------------------------------------------

. stmh survey if age<1 & year==23

         Failure _d: endevent==1
   Analysis time _t: (enddate-origin)/365.25
             Origin: time dob
  Enter on or after: time startdate
        ID variable: id
              Notes: age>5 trimmed
                     year>26 trimmed

Mantel–Haenszel estimate of the rate ratio
  comparing survey==1 vs. survey==0

------------------------------------------------------------------------
    Rate ratio         chi2         P>chi2          [95% conf. interval]
------------------------------------------------------------------------
         0.894         0.20         0.6558              0.547      1.461
------------------------------------------------------------------------

. stmh survey if age<5 & year==23

         Failure _d: endevent==1
   Analysis time _t: (enddate-origin)/365.25
             Origin: time dob
  Enter on or after: time startdate
        ID variable: id
              Notes: age>5 trimmed
                     year>26 trimmed

Mantel–Haenszel estimate of the rate ratio
  comparing survey==1 vs. survey==0

------------------------------------------------------------------------
    Rate ratio         chi2         P>chi2          [95% conf. interval]
------------------------------------------------------------------------
         0.933         0.15         0.6989              0.656      1.327
------------------------------------------------------------------------

.
. stmh survey if age==0 & year==24
```

```
         Failure _d: endevent==1
   Analysis time _t: (enddate-origin)/365.25
             Origin: time dob
  Enter on or after: time startdate
        ID variable: id
              Notes: age>5 trimmed
                     year>26 trimmed

Mantel-Haenszel estimate of the rate ratio
  comparing survey==1 vs. survey==0

-------------------------------------------------------------------------
    Rate ratio         chi2         P>chi2          [95% conf. interval]
-------------------------------------------------------------------------
         1.456         1.84         0.1754              0.843      2.515
-------------------------------------------------------------------------

. stmh survey if age<1 & year==24

         Failure _d: endevent==1
   Analysis time _t: (enddate-origin)/365.25
             Origin: time dob
  Enter on or after: time startdate
        ID variable: id
              Notes: age>5 trimmed
                     year>26 trimmed

Mantel-Haenszel estimate of the rate ratio
  comparing survey==1 vs. survey==0

-------------------------------------------------------------------------
    Rate ratio         chi2         P>chi2          [95% conf. interval]
-------------------------------------------------------------------------
         1.238         0.88         0.3493              0.791      1.936
-------------------------------------------------------------------------

. stmh survey if age<5 & year==24

         Failure _d: endevent==1
   Analysis time _t: (enddate-origin)/365.25
             Origin: time dob
  Enter on or after: time startdate
        ID variable: id
              Notes: age>5 trimmed
                     year>26 trimmed

Mantel-Haenszel estimate of the rate ratio
  comparing survey==1 vs. survey==0

-------------------------------------------------------------------------
```

```
    Rate ratio         chi2         P>chi2          [95% conf. interval]
------------------------------------------------------------------------
         0.976         0.02         0.8987              0.675      1.412
------------------------------------------------------------------------


.
. stmh survey if age==0 & year==25

         Failure _d: endevent==1
   Analysis time _t: (enddate-origin)/365.25
             Origin: time dob
  Enter on or after: time startdate
        ID variable: id
              Notes: age>5 trimmed
                     year>26 trimmed

Mantel-Haenszel estimate of the rate ratio
  comparing survey==1 vs. survey==0

------------------------------------------------------------------------
    Rate ratio         chi2         P>chi2          [95% conf. interval]
------------------------------------------------------------------------
         0.935         0.04         0.8443              0.477      1.832
------------------------------------------------------------------------

. stmh survey if age<1 & year==25

         Failure _d: endevent==1
   Analysis time _t: (enddate-origin)/365.25
             Origin: time dob
  Enter on or after: time startdate
        ID variable: id
              Notes: age>5 trimmed
                     year>26 trimmed

Mantel-Haenszel estimate of the rate ratio
  comparing survey==1 vs. survey==0

------------------------------------------------------------------------
    Rate ratio         chi2         P>chi2          [95% conf. interval]
------------------------------------------------------------------------
         0.980         0.04         0.8412              0.640      1.730
------------------------------------------------------------------------

. stmh survey if age<5 & year==25

         Failure _d: endevent==1
   Analysis time _t: (enddate-origin)/365.25
             Origin: time dob
  Enter on or after: time startdate
```

```
        ID variable: id
              Notes: age>5 trimmed
                     year>26 trimmed

Mantel-Haenszel estimate of the rate ratio
  comparing survey==1 vs. survey==0

-------------------------------------------------------------------------
    Rate ratio         chi2         P>chi2          [95% conf. interval]
-------------------------------------------------------------------------
         1.317         2.21         0.1369             0.915      1.895
-------------------------------------------------------------------------



.
. ***CHILD MORTALITY ESTIMATES (per 1,000 pop)
.
. **FARAFENNI
. keep if site=="FFN"

. * PERIODIC COMPARISONS

. stmh survey if age<4 & per==3

         Failure _d: endevent==1
   Analysis time _t: (enddate-origin)/365.25
             Origin: time (dob+365.25)
  Enter on or after: time startdate
        ID variable: id
              Notes: age>4 trimmed
                     year>26 trimmed

Mantel-Haenszel estimate of the rate ratio
  comparing survey==1 vs. survey==0

-------------------------------------------------------------------------
    Rate ratio         chi2         P>chi2          [95% conf. interval]
-------------------------------------------------------------------------
         0.779         2.30         0.1290             0.563      1.077
-------------------------------------------------------------------------

. stmh survey if age<4 & per==4

         Failure _d: endevent==1
   Analysis time _t: (enddate-origin)/365.25
             Origin: time (dob+365.25)
  Enter on or after: time startdate
        ID variable: id
              Notes: age>4 trimmed
```

```
                    year>26 trimmed

Mantel-Haenszel estimate of the rate ratio
  comparing survey==1 vs. survey==0

------------------------------------------------------------------------
    Rate ratio         chi2         P>chi2          [95% conf. interval]
------------------------------------------------------------------------
         0.701         3.10         0.0782               0.471      1.043
------------------------------------------------------------------------

. stmh survey if age<4 & per==5

         Failure _d: endevent==1
   Analysis time _t: (enddate-origin)/365.25
             Origin: time (dob+365.25)
  Enter on or after: time startdate
        ID variable: id
              Notes: age>4 trimmed
                     year>26 trimmed

Mantel-Haenszel estimate of the rate ratio
  comparing survey==1 vs. survey==0

------------------------------------------------------------------------
    Rate ratio         chi2         P>chi2          [95% conf. interval]
------------------------------------------------------------------------
         0.846         0.91         0.3401               0.599      1.194
------------------------------------------------------------------------


.
. *************************
. * ANNUAL COMPARISONS
. stmh survey if age<4 & year==21

         Failure _d: endevent==1
   Analysis time _t: (enddate-origin)/365.25
             Origin: time (dob+365.25)
  Enter on or after: time startdate
        ID variable: id
              Notes: age>4 trimmed
                     year>26 trimmed

Mantel-Haenszel estimate of the rate ratio
  comparing survey==1 vs. survey==0

------------------------------------------------------------------------
    Rate ratio         chi2         P>chi2          [95% conf. interval]
------------------------------------------------------------------------
         0.945         0.02         0.8761               0.463      1.928
```

```
------------------------------------------------------------------------

. stmh survey if age<4 & year==22

         Failure _d: endevent==1
   Analysis time _t: (enddate-origin)/365.25
             Origin: time (dob+365.25)
  Enter on or after: time startdate
        ID variable: id
              Notes: age>4 trimmed
                     year>26 trimmed

Mantel-Haenszel estimate of the rate ratio
  comparing survey==1 vs. survey==0

------------------------------------------------------------------------
    Rate ratio         chi2         P>chi2          [95% conf. interval]
------------------------------------------------------------------------
         1.107         0.07         0.7952              0.512      2.394
------------------------------------------------------------------------

. stmh survey if age<4 & year==23

         Failure _d: endevent==1
   Analysis time _t: (enddate-origin)/365.25
             Origin: time (dob+365.25)
  Enter on or after: time startdate
        ID variable: id
              Notes: age>4 trimmed
                     year>26 trimmed

Mantel-Haenszel estimate of the rate ratio
  comparing survey==1 vs. survey==0

------------------------------------------------------------------------
    Rate ratio         chi2         P>chi2          [95% conf. interval]
------------------------------------------------------------------------
         0.375         4.87         0.0274              0.151      0.929
------------------------------------------------------------------------

. stmh survey if age<4 & year==24

         Failure _d: endevent==1
   Analysis time _t: (enddate-origin)/365.25
             Origin: time (dob+365.25)
  Enter on or after: time startdate
        ID variable: id
              Notes: age>4 trimmed
                     year>26 trimmed
```

```
Mantel–Haenszel estimate of the rate ratio
  comparing survey==1 vs. survey==0

------------------------------------------------------------------------
    Rate ratio         chi2         P>chi2          [95% conf. interval]
------------------------------------------------------------------------
         0.631         1.03         0.3096              0.257      1.547
------------------------------------------------------------------------

. stmh survey if age<4 & year==25

         Failure _d: endevent==1
   Analysis time _t: (enddate-origin)/365.25
             Origin: time (dob+365.25)
  Enter on or after: time startdate
        ID variable: id
              Notes: age>4 trimmed
                     year>26 trimmed

Mantel–Haenszel estimate of the rate ratio
  comparing survey==1 vs. survey==0

------------------------------------------------------------------------
    Rate ratio         chi2         P>chi2          [95% conf. interval]
------------------------------------------------------------------------
         1.355         0.70         0.4035              0.662      2.772
------------------------------------------------------------------------

.

. **BASSE
. keep if site=="BAS"

. * PERIODIC COMPARISONS
. stmh survey if age<4 & per==4

         Failure _d: endevent==1
   Analysis time _t: (enddate-origin)/365.25
             Origin: time (dob+365.25)
  Enter on or after: time startdate
        ID variable: id
              Notes: age>4 trimmed
                     year>26 trimmed

Mantel–Haenszel estimate of the rate ratio
  comparing survey==1 vs. survey==0
```

```
------------------------------------------------------------------------
    Rate ratio         chi2         P>chi2          [95% conf. interval]
------------------------------------------------------------------------
         0.660         8.30         0.0040             0.496       0.877
------------------------------------------------------------------------

. stmh survey if age<4 & per==5

         Failure _d: endevent==1
   Analysis time _t: (enddate-origin)/365.25
             Origin: time (dob+365.25)
  Enter on or after: time startdate
        ID variable: id
              Notes: age>4 trimmed
                     year>26 trimmed

Mantel-Haenszel estimate of the rate ratio
  comparing survey==1 vs. survey==0

------------------------------------------------------------------------
    Rate ratio         chi2         P>chi2          [95% conf. interval]
------------------------------------------------------------------------
         0.815         2.39         0.1222             0.629       1.057
------------------------------------------------------------------------

.

. * ANNUAL COMPARISONS
. stmh survey if age<4 & year==21

         Failure _d: endevent==1
   Analysis time _t: (enddate-origin)/365.25
             Origin: time (dob+365.25)
  Enter on or after: time startdate
        ID variable: id
              Notes: age>4 trimmed
                     year>26 trimmed

Mantel-Haenszel estimate of the rate ratio
  comparing survey==1 vs. survey==0

------------------------------------------------------------------------
    Rate ratio         chi2         P>chi2          [95% conf. interval]
------------------------------------------------------------------------
         0.674         1.68         0.1949             0.370       1.229
------------------------------------------------------------------------

. stmh survey if age<4 & year==22
```

```
         Failure _d: endevent==1
   Analysis time _t: (enddate-origin)/365.25
             Origin: time (dob+365.25)
  Enter on or after: time startdate
        ID variable: id
              Notes: age>4 trimmed
                     year>26 trimmed

Mantel–Haenszel estimate of the rate ratio
  comparing survey==1 vs. survey==0

-------------------------------------------------------------------------
    Rate ratio         chi2        P>chi2          [95% conf. interval]
-------------------------------------------------------------------------
         0.470         4.65        0.0311              0.233      0.949
-------------------------------------------------------------------------

. stmh survey if age<4 & year==23

         Failure _d: endevent==1
   Analysis time _t: (enddate-origin)/365.25
             Origin: time (dob+365.25)
  Enter on or after: time startdate
        ID variable: id
              Notes: age>4 trimmed
                     year>26 trimmed

Mantel–Haenszel estimate of the rate ratio
  comparing survey==1 vs. survey==0

-------------------------------------------------------------------------
    Rate ratio         chi2        P>chi2          [95% conf. interval]
-------------------------------------------------------------------------
         0.967         0.02        0.8971              0.582      1.606
-------------------------------------------------------------------------

. stmh survey if age<4 & year==24

         Failure _d: endevent==1
   Analysis time _t: (enddate-origin)/365.25
             Origin: time (dob+365.25)
  Enter on or after: time startdate
        ID variable: id
              Notes: age>4 trimmed
                     year>26 trimmed

Mantel–Haenszel estimate of the rate ratio
  comparing survey==1 vs. survey==0

-------------------------------------------------------------------------
```

```
    Rate ratio         chi2         P>chi2          [95% conf. interval]
------------------------------------------------------------------------
         0.589         2.37         0.1235              0.298      1.164
------------------------------------------------------------------------

. stmh survey if age<4 & year==25

         Failure _d: endevent==1
   Analysis time _t: (enddate-origin)/365.25
             Origin: time (dob+365.25)
  Enter on or after: time startdate
        ID variable: id
              Notes: age>4 trimmed
                     year>26 trimmed

Mantel–Haenszel estimate of the rate ratio
  comparing survey==1 vs. survey==0

------------------------------------------------------------------------
    Rate ratio         chi2         P>chi2          [95% conf. interval]
------------------------------------------------------------------------
         1.620         3.14         0.0762              0.946      2.774
------------------------------------------------------------------------


.
end of do-file

. log close
      name:  <unnamed>
       log:  C:\Users\mjasseh\.....\Childhood Mortality Indicators by
Period - All - Stata 17.smcl
  log type:  smcl
 closed on:   5 May 2022, 16:13:12
--------------------------------------------------------------------------
```
