# Supplementary material for: Assessment of the consistency of health and demographic surveillance and household survey data: A demonstration at two HDSS sites in The Gambia
Source: PLoS One. 2022 Jul 13;17(7):e0271464. doi: 10.1371/journal.pone.0271464 (PMC9278757; doi:10.1371/journal.pone.0271464)
Supplement: S5 File — (DOCX) [file pone.0271464.s005.docx]

**Notes on Figure 1**

Figure 1 was revised using publicly available maps from “https://d-maps.com” and “https://commons.wikimedia.org” with the following source details:

1. Political Map of Africa (labelled “A” in Figure 1):

<https://d-maps.com/carte.php?num_car=737&lang=en>

<https://d-maps.com/m/africa/afrique/afrique10.gif> (image file).

1. Political Map of West Africa (labelled “B” in Figure 1):

<https://d-maps.com/carte.php?num_car=753&lang=en>

<https://d-maps.com/m/africa/west/west06.gif> (image file).

Permission for use of both maps has been granted by the owner of d-map.com and attached as supporting documents in the submission. They have also been cited in the list of references.

1. The Gambia (with Districts):

<https://commons.wikimedia.org/wiki/File:Gambia_districts.png>

<https://en.wikipedia.org/wiki/User_talk:Rarelibra/Maps2#/media/File:Gambia_districts.png> (image file)

The terms of use are indicated in this link:

<https://commons.wikimedia.org/wiki/File:Gambia_districts.png#filelinks>

and states:

“I, the copyright holder of this work, release this work into the [**public domain**](https://en.wikipedia.org/wiki/en:public_domain). This applies worldwide. In some countries this may not be legally possible; if so:
*I grant anyone the right to use this work****for any purpose****, without any conditions, unless such conditions are required by law”.*
